# Pressurized IntraPeritoneal Aerosol Chemotherapy (PIPAC)-directed treatment of peritoneal metastasis in end-stage colo-rectal cancer patients

**DOI:** 10.1515/pp-2020-0109

**Published:** 2020-05-15

**Authors:** Signe Bremholm Ellebæk, Martin Graversen, Sönke Detlefsen, Lars Lundell, Claus W. Fristrup, Per Pfeiffer, Michael B. Mortensen

**Affiliations:** Surgical department, Odense Universitetshospital, Odense Denmark; Department of Pathology, Odense Universitetshospital, Odense, Denmark; Division of Surgery, CLINTEC, Karolinska Institutet, Stockholm, Sweden; Department of Oncology, Odense Universitetshospital, Odense, Denmark; Upper GI and HPB Section, Department of Surgery, Odense Universitetshospital, Odense, Denmark

**Keywords:** colon cancer, complications, intraperitoneal chemotherapy, peritoneal metastasis, Pressurized IntraPeritoneal Aerosol Chemotherapy (PIPAC), rectum cancer

## Abstract

**Background:**

Pressurized IntraPeritoneal Aerosol Chemotherapy (PIPAC) represents a novel approach to intraperitoneal chemotherapy. Hereby results, obtained with PIPAC in patients with advanced peritoneal metastasis (PM) from colorectal cancer (CRC), are presented.

**Methods:**

Data from CRC patients (*n* = 24) included in the prospective PIPAC-OPC1 and PIPAC-OPC2 trials are reported. Oxaliplatin 92 mg/m^2^ was administered at 4-6-week intervals. A CE certified nebulizer was used to aerosolize the chemotherapeutics. Outcome criteria were objective tumor response, survival and adverse events.

**Results:**

Retrospective analysis of 74 PIPAC procedures carried out in 24 consecutive patients with PM from CRC included from October 2015 to February 2019. Five patients had still the primary tumor in situ, and 22 patients had received palliative systemic chemotherapy. Nineteen patients completed more than two PIPAC procedures, and objective tumor response according to the histological Peritoneal Regression Grading Score (PRGS) was observed in 67% of the patients, while 21% had stable disease. Four patients (21%) had complete response (mean PRGS = 1 and negative cytology). We recorded a median survival of 37.6 (range 7.3–48.9) months from the time of PM diagnosis, whereas it was 20.5 (range 0.13–34.7) months following the first PIPAC session. Minor postoperative complications were noted, and few were considered causally related to the PIPAC treatment. However, two cases of severe postoperative complications were recorded (urosepsis and iatrogenic bowel perforation).

**Conclusions:**

PIPAC with low-dose oxaliplatin can induce objective tumor regression in selected patients with advanced PM from colorectal cancer.

## Introduction

Cancer of the colon and rectum is one of the most common cancer diseases worldwide and remains the second most common cause of cancer death in Western countries [1]. Metastatic disease is the leading cause of mortality in colorectal cancer (CRC) patients, and peritoneal metastasis (PM) is the second most common site of recurrence, accounting for 25–35% [2, 3]. Traditionally, patients with PM have a very poor prognosis and a short life expectancy due to limited treatment options and poor performance status [4]. During the last decades, significant changes have been introduced in the management of CRC patients with PM, which include cytoreductive surgery (CRS) and hyperthermic intraperitoneal chemotherapy (HIPEC) added to systemic chemotherapy. Five-year overall survival rates have improved significantly when CRS plus HIPEC is offered in selected patients [5, 6]. For patients with non-resectable CRC-PM, palliative systemic chemotherapy is the primary treatment strategy, but the median survival is short [7, 8]. A significant therapeutic obstacle is raised by the fact that systemic chemotherapy is less effective against PM, mainly due to pharmacokinetic limitations to reach the PM, combined with poor peritoneal vascularization. Pressurized IntraPeritoneal Aerosol Chemotherapy (PIPAC) is a novel, safe and feasible technique delivering cytotoxic drugs into the abdominal cavity as an aerosol under pressure [9, 10]. PIPAC-directed treatment may provide an objective tumor response in a large subset of patients with PM and can often be administered as an outpatient procedure [9, 11]. However, specific data on the effect of PIPAC-directed treatment in CRC-PM, based on an objective and validated model for response evaluation, are still lacking.

With this study, we present the results of PIPAC in a consecutive cohort of patients having PIPAC for PM from CRC. The main outcome was to evaluate the objective tumor response based on repeated peritoneal biopsies according to the Peritoneal Regression Grading Score (PRGS) [12, 13]. Secondary outcomes included median overall survival after the diagnosis and after the first PIPAC-directed treatment, ascites formation, peritoneal lavage cytology and treatment related adverse reactions.

## Patients and methods

Data from patients with CRC-PM included in the prospective PIPAC-OPC1 and PIPAC-OPC2 trials are reported. The PIPAC-OPC1 trial has been completed and published [9] whereas the PIPAC-OPC2 trial is ongoing [14]. CRC-PM was documented through radiology, histology or cytology, patients were discussed at a dedicated Multi-Disciplinary Tumor conference (MDT), and no patients were eligible for CRS and HIPEC according to national guidelines. Patients with a maximum of one extraperitoneal metastasis were included and females had to be post-menopausal. Patients were older than 18 years with an Eastern Cooperative Oncology Group performance status of less than 2. The exclusion criteria of the study were as follows: gastrointestinal tract obstruction, a history of allergic reactions to oxaliplatin, renal impairment (GFR<40 mL/min), myocardial insufficiency (NYHA class>2), impaired liver function (bilirubin>1.5 upper normal limit) or inadequate haematological function (ANC<1.5 × 10^9^ /L or plates<100 × 10^9^ /L).

### PIPAC

PIPAC-directed treatment with oxaliplatin 92 mg/m^2^ in 150 mL dextrose was performed in the setting of a diagnostic laparoscopy, as described in detail previously [15, 16]. Patients were scheduled for three PIPAC procedures at intervals of four to six weeks (six to seven weeks if combined with systemic chemotherapy as bidirectional treatment).

For safe open access to the abdominal cavity, all procedures were preceded by percutaneous ultrasound performed by the surgeon and the patients received prophylactic antibiotics. The extent of PM was evaluated according to Sugarbaker’s Peritoneal Cancer Index (PCI) [5], ascites or peritoneal lavage fluid was evacuated and peritoneal biopsies from each affected quadrant of the abdominal cavity were obtained. The biopsy sites were marked by metal clips allowing repeated biopsies from the same site during the following PIPAC procedures. A CE certified nebulizer (CapnoPen^®^, Capnomed, Villingendorf, Germany) was used to aerosolize the chemotherapeutics at a flow rate of 0.7 mL/s and a maximum pressure of 300 PSI. Due to a protocol amendment in the PIPAC-OPC2 trial, 13 of the patients were treated by standard PIPAC-directed treatment (30 minutes of simple diffusion), while seven of the patients were treated by ePIPAC. At ePIPAC, the same steps regarding safety and chemotherapy administration were followed, but after intraperitoneal delivery of chemotherapy, the Ultravision generator (Ultravision, Alesi Surgical Ltd., UK) was turned on, and electrostatic precipitation was performed until the aerosol was cleared completely by visual inspection Following evacuation of CO_2_ through a closed air waste system, the patients were closed according to departmental guidelines. The patients were discharged if pain was adequately relieved and organ functions were normal. Patients had access to a hotline telephone number enabling immediate contact to the surgical department in case of emergencies or unexpected toxicity. Patients were routinely contacted after 14 days by the principal investigator or a dedicated study nurse.

Thirty days surgical complications were graded according to the Clavien-Dindo [17] classification and adverse events were graded according to the CTCAE version 4.0 [18].

A contrast-enhanced multi-slice CT of the thorax and abdomen was performed after three PIPAC treatments. The PIPAC-directed treatment was continued for another three courses if the CT did not show extra-peritoneal disease progression, if the patient had no unacceptable treatment related adverse reactions and had responded or stabile disease according to the PRGS.

### Evaluation of treatment response

The response to PIPAC-directed treatment was based on the histological assessment of repeated peritoneal biopsies and cytological assessment of ascites/peritoneal lavage fluid retrieved before each PIPAC procedure.

Each peritoneal biopsy was fixed in formalin and embedded in paraffin. Three step sections with a distance of 15–50 µm between each section were cut from the paraffin-embedded tissue blocks and stained with H&E, followed by a section immunostained for EpCAM and a final series of three step sections stained with H&E. The slides were analyzed by the gastrointestinal study pathologist who was involved in the planning and conduction of the project (SDE). Besides, some cases were examined by another dedicated gastrointestinal pathologist. The PRGS was used for evaluation of the histological regression [12, 13]. A decrease of the mean PRGS during the course of therapy was considered as response to treatment, while the mean PRGS remained unchanged in stable disease. Complete response was defined as PRGS = 1 in all biopsies from respective abdominal quadrants, and lack of malignant cells at peritoneal cytology.

Peritoneal lavage was performed at the start of each procedure by injecting 500 mL saline, if no ascites was present. A total of 150 mL of ascites or peritoneal lavage fluid was then retrieved and analyzed for cancer cells by conventional cytology including preparation of one or two cell blocks for immunocytochemical analyses, if needed. A five-tied score was used for cytological evaluation: malignant cells, suspicious cells, atypical cells, no malignant cells, other. Malignant and suspicious cells were defined as positive cytology.

### Follow-up

Patients were followed until death or 01.05.2019 (data processing date).

### Statistics

Values are given as means or medians where appropriate. The survival analyses used traditional Kaplan–Meier plots. Otherwise only descriptive statistics have been applied. The statistical software Stata, version 13 (Stata Corp, Texas, USA) was used for the statistical analyses.

### Ethics

The studies have been conducted according to predefined protocols and the Helsinki declaration. The recommendations developed by “The Strengthening the Reporting of Observational Studies in Epidemiology (STROBE) Initiative” have been followed. Oral and written informed consent was obtained from each patient. The study protocols were approved by The Regional Committees on Health Research Ethics for Southern Denmark (Project-ID: S-20140211 and S-20160100), the Danish Medicines Agency (Code number: 2016083464) and the Danish Data Protection Agency (14/52603 and 16/23653) and registered at www.clinicaltrials.gov (ClinicalTrials.gov identifier: NCT02320448 and NCT03287375) and the European Clinical Trials Database (EudraCT) number 2016-003394-18.

## Results

Patients were included from October 2015 to February 2019 and the last PIPAC was completed in April 2019. During this period 24 patients with PM from CRC were scheduled for PIPAC therapy whereupon 75 PIPAC procedures were completed. The preoperative and procedure related patient characteristics are summarized in Table 1, but only patients amenable to undergo more than 1 treatment session were included in the final analyses.

**Table 1:** Baseline demographic data.

Number of patients	24
Number of procedures	75
Age: years, median (range)	64 (40–80)
Performance status	
0	7 (29%)
1	14 (58%)
2	3 (13%)
Gender	
M/F	13/11
Chemotherapy	
Neoadjuvant^a^	4 (17%)
Adjuvant^b^	10 (42%)
Palliative^c^	22 (91%)
Bidirectional treatment^d^	3 (12.5%)
PCI score (median, range)	
PCI when ≥ 11 regions evaluated (*n* = 16)	14.8 (1–30)
PCI when<11 regions evaluated (*n* = 8)	2.6 (1–8)
PCI total	10.7 (1–30)
Ascites	
Yes (%)	7 (29%)
Median, range (mL)	50 (10–2700)

^a^Four patients received neoadjuvant chemotherapy prior to primary colorectal cancer surgery. ^b^Ten patients received adjuvant chemotherapy after primary colorectal cancer surgery. ^c^Two patients did not want to receive systemic chemotherapy. ^d^Tree patients received bidirectional treatment (PIPAC and systemic palliative chemotherapy).

All patients had colorectal adenocarcinoma, five patients had mucinous type and one patient had signet ring cell carcinoma. Nineteen patients had undergone primary resection previously, while the remaining five patients had their tumor in situ when PM was diagnosed. Twenty-two patients had received palliative systemic chemotherapy prior to the PIPAC-directed treatment, and three of these patients received bidirectional palliative chemotherapy during the entire PIPAC treatment period. Fourteen patients had ended first-line chemotherapy treatment, six patients the second-line and two patients the third-line before enrolling the course of PIPAC treatment.

A total number of 75 PIPAC procedures were given (13 e-PIPAC, 62 PIPAC) with a median operating time of 90 minutes (range 44–155) and 71 minutes (range 52–110) for PIPAC and e-PIPAC, respectively. No intraoperative complications were recorded.

**Figure 1:**
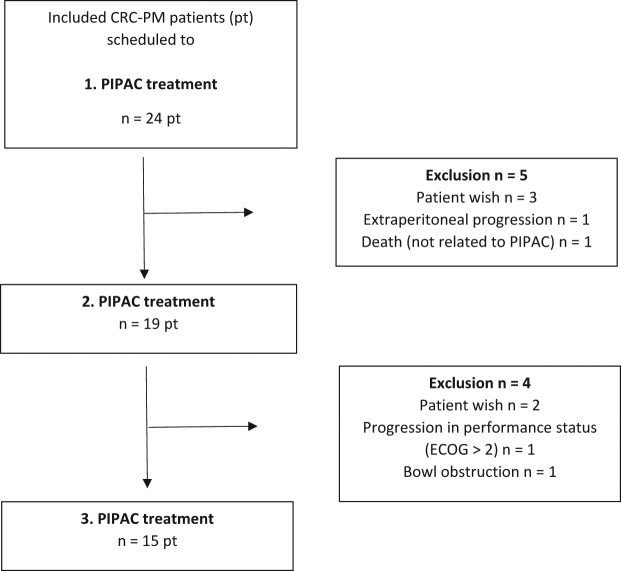
Flow chart of the included patients.

Nineteen patients completed two PIPAC procedures and 15 patients completed three PIPAC procedures (per protocol group). Seven patients had more than three procedures and one had seven treatments. The details behind the withdrawals are given in Figure 1.

**Figure 2:**
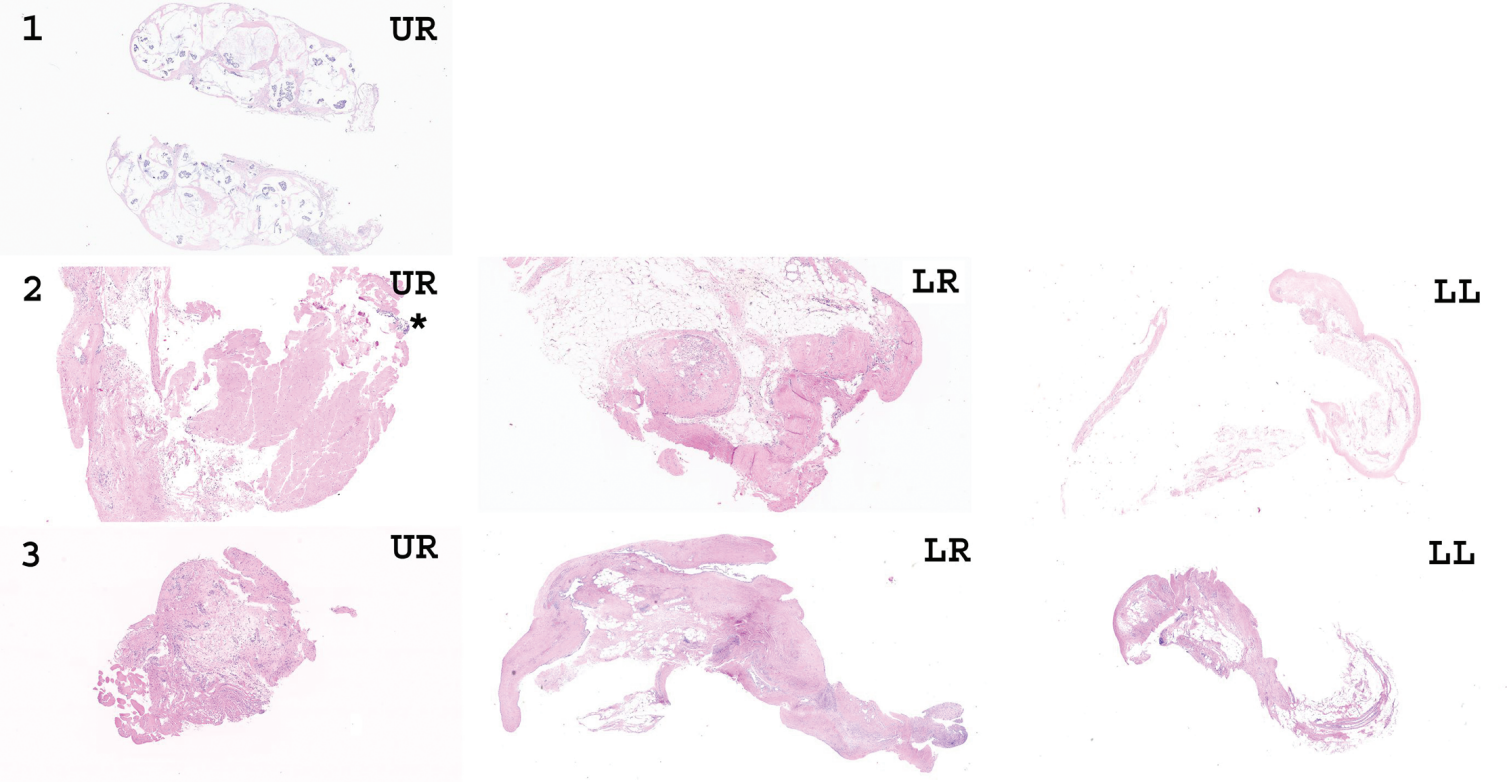
Histological images of peritoneal quadrant biopsies taken prior to PIPAC treatment 1, 2 and 3 for patient with complete response according to PRGS. First row (1): At PIPAC 1, it was only possible to obtain one quadrant biopsy from the upper right quadrant (UR), showing mucinous adenocarcinoma without any signs of regression (PRGS score 4). Second row (2): At PIPAC 2, a tiny focus of adenocarcinoma was present (asterix) in the biopsy from the UR, while the quadrant biopsies from the lower right (LR) and lower left (LL) quadrants only showed regression without malignancy (highest PRGS score 2, average PRGS score 1.33). Third row (3): At PIPAC 3, the quadrant biopsies from UR, LR and LL all were without malignancy. Instead, regressive features were present (average PRGS score 1).

As a response to the first PIPAC procedure, histological regression was seen in thirteen patients out of those 19 who completed 2 sessions (68%), with the corresponding intention to treat (ITT *n* = 24) figures being 54%, while four patients (21%, ITT 17%) had stable disease. After the second PIPAC session histological regression was noted in ten patients (67%, ITT 42%) and four (27%, ITT 17%) had stable disease (Table 2). Figure 2 shows histological images of all quadrant biopsies taken from patient number 1.

**Table 2:** Peritoneal Regression Grading Score (PRGS 1–4), at baseline (i.e. before PIPAC 1) compared to the situation immediately before the third PIPAC procedure (*n* = 15).

Patient no.	PIPAC 1 PRGS (highest/mean)	PIPAC3 PRGS (highest/mean)	Histological response^a^
1	4/2.0	1/1.0	+
2	2/2.0	2/1.5	+
3	1/1.0	1/1.0	±
4	2/1.5	2/1.5	±
5	2/2.0	1/1.0	+ (CR)
6	2/1.75	2/1.25	+
7	3/2.0	2/1.67	+
8	2/1.0	1/1.0	+
9	3/3.0	1/1.0	+ (CR)
10	3/2.0	3/2.0	±
11	2/1.25	1/1.0	+ (CR)
12	2/1.33	1/1.0	+ (CR)
13	2/1.5	4/2.5	–
14	1/1.0	1/1.0	±
15	2/2.0	2/1.25	+

^a^+, regression; –, progression; ±, stable disease according to PRGS; CR, complete response (PRGS 1+non-malign cytology).

Four patients (21%) had complete response (mean PRGS = 1 and negative cytology) as illustrated in Figure 2.

Seven patients (21%) had ascites at the time of the first PIPAC procedure which was reduced to four patients at the end of the third session. The amount of ascites was reduced from the first PIPAC (median 50 mL, range 10–2700 mL) to the last PIPAC (median 40 mL, range 20–200 mL).

Among the 15 patients who completed all 3 PIPAC procedures had the peritoneal lavage fluid analyzed for malignant cells and five of these (33%) converted from positive to negative cytology, while two patients (13%) converted from negative to positive cytology.

**Figure 3:**
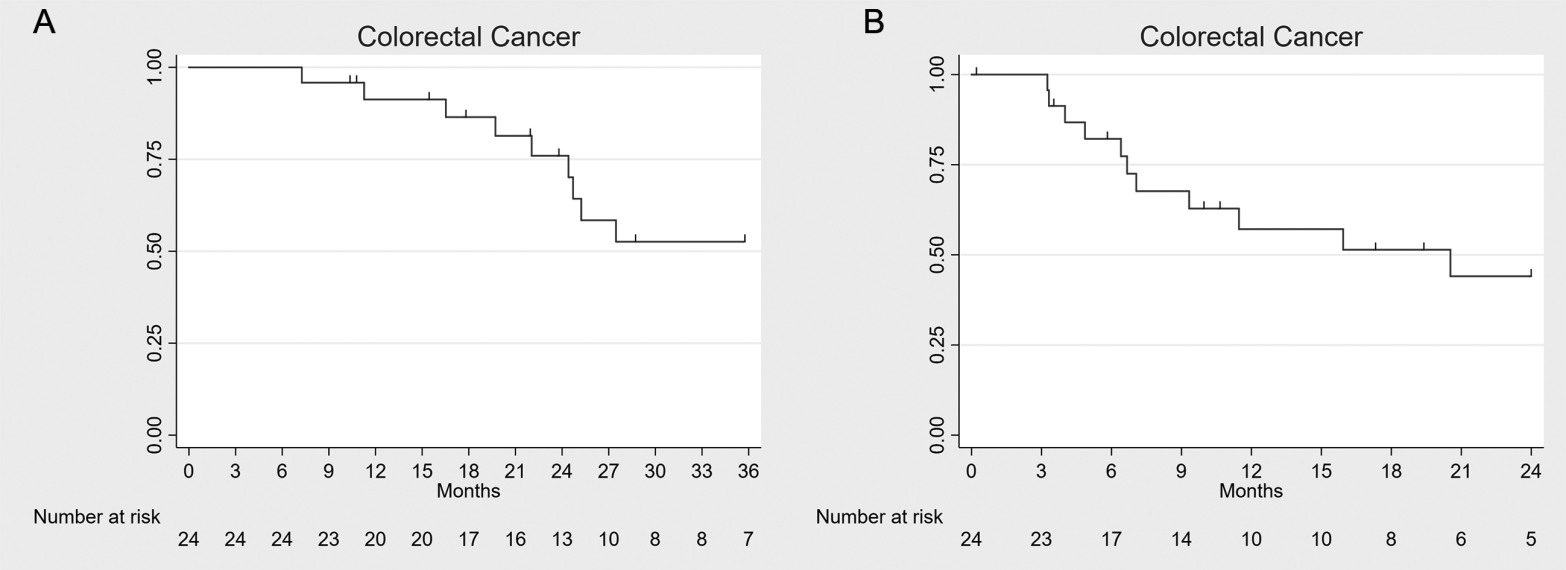
Kaplan–Meier survival plots in colorectal cancer patients with peritoneal metastasis subjected to PIPAC treatment. Survival from diagnosis of PM (A) and from the first PIPAC procedure (B).

We recorded a median survival of 37.6 (range 7.3–48.9) months from the time of PM diagnosis (Figure 3A), whereas it was 20.5 (range 0.1–34.7) months following the start of the first PIPAC session (Figure 3B).

Median follow-up was 28.6 months and thirteen patients were alive at the end of the follow-up period. None of these patients fulfilled the national guidelines for CRS/HIPEC.

All postoperative adverse events/reactions were recorded. Most of the reactions were scored as mild to moderate adverse events. We observed minor reversible neuropathy, urinary retention, nausea and pain, which probably could be related to the PIPAC-directed treatment. One case of severe adverse event occurred in the form of urosepsis from urinary retention due the PIPAC treatment and a life-threatening adverse event was caused by an iatrogenic perforation of the jejunum which required reoperation with primary suture on the first postoperative day.

## Discussion

Restricted amount of data is available on the value of adding intraperitoneal chemotherapy in advanced PM from CRC. In this setting the present results are of interest showing a significant number of patients responding objectively to repeated PIPAC treatment with oxaliplatin in a dose of 92 mg/m^2^ per session. These results were obtained in chemotherapy-resistant CRC peritoneal metastases. Nineteen patients completed two PIPAC procedures and 15 patients completed three PIPAC procedures. Seven patients had more than three procedures and one had seven treatments.

In the phase of critical evaluation of this new technology means to obtain a valid objective response is critical. We found at total PCI score of 10.7 (range 1–30) at the index PIPAC procedure. The PCI score is valuable to describe the study population at the index PIPAC procedure, but the PCI score is a suboptimal tool for evaluation of the response to PIPAC, as the PCI cannot differ between macroscopic progression and treatment induced fibrosis. Secondly, several areas may not be accessible for scoring (Table 1). This is particularly so when the only approach to the abdominal cavity is through the laparoscope. Ongoing and future research has to clarify the most optimal evaluation strategy. Currently, the response evaluation strategy was based on repeated histological biopsies for PRGS assessment and peritoneal lavage cytology. Hereby we found that histological regression (decline in PRGS score) was seen in thirteen patients (68%) while four (21%) had stable disease after the first PIPAC procedure. After the second PIPAC session histological regression was noted in ten patients (67%) and four (27%) had stable disease. Noteworthy is that in such a demanding clinical situation we obtained complete response (mean PRGS = 1 and negative cytology) in four patients (21%) all of whom were alive after a median follow-up of 28.6 months. A significant proportion of lavage positive patients converted to become non-malignant, an observation which mandates additional studies applying more advanced technologies to document its clinical relevance [19]. Another aspect, with alleged relevance for the palliative management of these advanced CRC patients, was reflected by our observation that PIPAC substantially reduced ascites formation. Similar observations have been made when using PIPAC-directed therapy in PM for other diseases [20]. When we followed all our 24 patients, we recorded a median survival of 37.6 (range 10.2–47.0) months from the time of PM diagnosis, whereas it was 20.5 (range 0.13–34.7) months following the start of the first PIPAC session. These figures have to be put into perspective knowing that the prognosis for patients with CRC, combined with advanced PM, is poor with a reported median survival of only 5 months (95% CI 3–7 months) with best supportive care [7, 21]. Moreover, the present cohort of patients can, with regard to the extent and severity of disease burden, best be compared to those exposed to aborted CRS/HIPEC procedures. The primary reason behind aborted CRS is widespread abdominal disease, why one can expect those patients to suffer a rapidly progressive and lethal clinical course [22]. In the series by Rodt et al., the median survival in corresponding situations was 12.7 months, whereas others have reported that only about half of the patients who underwent aborted CRS procedures went on to receive palliative chemotherapy associated with a survival of only 8 months [23]. The Dutch group likewise observed that similar patients fared a dismal prognosis with a median survival of 11.2 months in these situations even for patients treated with palliative chemotherapy compared to only 2.7 months for those with best supportive care alone [21]. Accordingly, the present survival figures are encouraging and adopt well to the hitherto limited data available on PIPAC treatment in advanced CRC clinical stages [10]. These results, observed in a selected group of very advanced primary and recurrent CRC, add further evidence to suggest that PIPAC meets the clinical need for new and better therapies urgently requested in patients with such a severe cancer disease. Corresponding data justify prospective comparative clinical studies of PIPAC as a palliative therapy in CRC PM patients, who are not candidates for CRS ± HIPEC. The future prospects for HIPEC in CRC-PM patients can, however, be challenged based on results recently presented from large clinical randomized trials [24, 25]. Therefore, attention will eventually move towards alternative tools to administer intraperitoneal chemotherapy. The feasibility and safety of PIPAC have now reached the status when it can be more widely used in clinical practice although preferably within the framework of research protocols [9, 15, 26]. PIPAC also needs to be evaluated as an alternative to HIPEC as an adjunct to CRS and as an adjuvant therapeutic concept in high risk CRC patients submitted to a curative resection. Ongoing and future trials have to clarify the role of this therapeutic concept in these settings [27]. Current and future challenges for PIPAC has recently been surveyed [28, 29].

The dose of oxaliplatin currently applied during PIPAC was determined arbitrarily and copied the drug concentration in the aerosol as practiced in the HIPEC perfusate [30]. Determination of the optimal dose via a dose-finding study is currently under way. In terms of side effects only pain scores increased slightly, although frequent this negative effect was transitory. Transient abdominal pain might be explained by the chemical peritonitis induced by PIPAC with oxaliplatin. This observation may be of particular clinical relevance since the control of this pain may be critical for the completion of these sessions in an outpatient setting [11]. Some of the patients experienced reversible urinary retention, properly due to pain and the chemical peritonitis induced by PIPAC. Classical side-effects of systemic chemotherapy such as mucositis, nausea/ vomiting, diarrhea, paresthesia, cutaneous symptoms and alopecia were not reported by the patients.

## Conclusions

PIPAC with oxaliplatin can induce objective tumor regression in the majority of selected patients with advanced PM from CRC offering survival prospects that are encouraging but need to be further explored.
